# Potential shared gene signatures and molecular mechanisms between recurrent pregnancy loss and ovarian cancer

**DOI:** 10.3389/fonc.2024.1445502

**Published:** 2024-11-14

**Authors:** Yan Wang, Yan Cai, Jiadong Chen, Wenzhe Shen, Jianqing Zhu, Qiming Wang

**Affiliations:** ^1^ Gynecology, Women and Children’s Hospital of Ningbo University, Ningbo, China; ^2^ Gynecological Oncoloy Department, Zhejiang Cancer Hospital, Hangzhou, China

**Keywords:** common gene, ovarian cancer, recurrent pregnancy losses, prognostic risk score system, immunotherapy

## Abstract

**Background:**

Ovarian cancer (OV) is the second most prevalent gynecological tumor. Recurrent pregnancy loss (RPL) refers to two or more spontaneous abortions. However, the molecular mechanisms underlying both OV and RPL remain poorly understood. This article focuses on the exploration of the common genetic characteristics of OV and RPL and their molecular mechanisms.

**Methods:**

The 71 differentially expressed genes associated with RPL and 1427 genes associated with OV survival were analyzed, among which 7 common genes were both important in the pathogenesis of RPL and OV. Then stepAIC analysis was performed to simplify the model and decrease the number of genes, which yielded a final set of 5 prognostic genes with coefficients to construct a prognostic risk scoring system. Univariate and multivariate Cox analyses were conducted to verify the independent prognostic factor for OV patients. GSEA and GO analysis results showed enriched biological pathways in the high/low risk groups, thereby revealing their biological characteristics. The effect of immunotherapy is better in LR patients. There was a significantly higher enrichment score of stemness and higher tumor aneuploidy score in the HR group.

**Results:**

A five-gene prognostic risk model provided a more accurate prognosis for OV, and this prognostic score system was validated using two external cohorts. The risk score was an independent prognostic index for OV patients. Based on levels of ICs, immune cell infiltration, and predicted response, low risk OV patients were more likely to benefit from immunotherapies.

**Conclusions:**

The 5-gene risk model can predict the prognosis of OV patients, which can draw the attention of clinicians and help stratify patients into high and low risk groups for management.

## 1 Introduction

Ovarian cancer (OV) is the second most prevalent gynecological malignancy, only after cervical cancer and stands as the eighth primary contributor to female cancer-related mortality. Each year, there are 239,000 new cases and 152,000 deaths worldwide (1). Among the multiple histological and molecular subtypes of OV, 95% of cases are epithelial, while 5% are non-epithelial cancers, mainly including sex-cord stromal cancers and germ cell, as well as rare sarcomas of the ovary and small cell carcinomas (2). Despite the availability of standardized treatments, such as comprehensive staging surgery and platinum-taxane combination chemotherapy, the mortality rate of OV remains high, and the prognosis remains unfavorable. Ovarian cancer is highly malignant, uncontrolled exponential growth of a malignant cancerous tumor is mathematically identically to the model of the growth of bacterial colonies (3). Even in resource-rich countries like Canada and the United States, the overall survival (OS) of OV has changed little over the decades, remaining at only 47% five years after diagnosis (4). Molecular targeted anti-cancer therapies and immunotherapies are mostly in the clinical trial, and more effective treatment strategies are required for OV.

According to the guidelines from the American Society for Reproductive Medicine (ASRM) and the European Society of Human Reproduction and Embryology (ESHRE), recurrent pregnancy loss (RPL) refers to two or more natural miscarriages (5, 6). Its etiology is intricate, with chromosomal abnormalities being the predominant factor. The management of RPL in clinical settings presents considerable difficulties, and the cause remains unknown for some patients. Unlike spontaneous miscarriage, RPL requires medical intervention and regular medical monitoring during pregnancy. Nevertheless, achieving a complete cure for RPL remains a formidable task. RPL may be characterized by vaginal bleeding and lower abdominal pain after a missed period, but in some patients, it may be asymptomatic. Autoimmune disorders and structural uterine abnormalities are associated with RPL, but it remains unclear why these conditions impact only certain pregnancies rather than all. More than 50% of women do not exhibit any recognized risk factors for miscarriage (7, 8).

The incidence of OV is associated with women’s reproductive status. Delaying childbirthing age and increasing parity are both important protective factors against ovarian cancer (9). A full-term pregnancy can reduce a woman’s risk of cancer, and an incomplete pregnancy can also provide some protection. Therefore, OV and RPL may share common molecular pathogenesis and thus increase the risk of malignant tumors in women. Studies suggest that one possible shared mechanism is the insufficient progesterone secretion in the female corpus luteum, leading to endocrine disorders and weakening the repressing effect of progesterone on the future development of OV (10). Evidence suggests that multiple miscarriages in women may enhance the risk of epithelial OV, due to common molecular mechanisms (11). Thereby, a comprehensive understanding of the potential molecular mechanisms or molecular pathways of OV and RPL is essential for the identification of molecular or genetic therapeutic targets. This will help clinicians effectively treat and manage these two conditions, thus providing much-needed relief to patients who have endured prolonged suffering.

Rapid advances in genomics and molecular biology have allowed us to quickly understand the genetic profiles of various diseases. Through the comparison of genes between diseased individuals and healthy counterparts, molecular targets for tumors can be pinpointed, thereby providing advantages to patients. Despite the availability of rigorous treatments, treatment strategies and prognosis for patients with OV and RPL require additional refinement. This investigation employed GEO and TCGA datasets to identify common genes between OV and RPL and elucidate the shared molecular mechanisms. Hence, we established an OV prognostic model based on 5 common genes and stratified clinical subjects into high risk (HR) and low risk (LR) groups. Through in-depth analysis of survival and immunotherapy aspects, we revealed the clinical significance of the prognostic risk model and provided treatment directions for OV patients.

## 2 Material and methods

### 2.1 Data collection

One RPL dataset, GSE165004, was downloaded from the NCBI Gene Expression Omnibus (GEO; https://www.ncbi.nlm.nih.gov/geo/). The GSE165004 dataset was generated using the GPL16699 Agilent-039494 SurePrint G3 Human GE v2 8x60K Microarray 039381 (Feature Number version). The GSE165004 dataset consisted of 24 RPL samples and 24 normal samples.

Three OV datasets, GSE63885, GSE26193, and TCGA OV were retrieved from the GEO and the UCSC Xena website (https://xena.ucsc.edu/). GSE63885 and GSE26193 datasets were obtained through GPL570 (HG-U133 Plus 2). TCGA OV dataset consisted of 353 tumor samples. GSE63885 and GSE26193 datasets, as external validation cohorts, encompassed 75 OV samples and 107 OV samples, respectively.

GEO data were preprocessed using the R package “GEOquery”. Gene probes were annotated using gene symbols, and probes that either did not match any gene symbol or matched multiple gene symbols were removed. Ultimately, the gene expression values for repeated gene symbols were calculated as the maximum value.

### 2.2 Common gene screening and analysis

The RPL DEGs were screened out using the R package “limma” in the GSE165004 dataset, with |LogFC| > 1 and P < 0.05 as the threshold.

Then, the prognostic genes of OV were estimated through univariate Cox analysis (P<0.05). The common genes were obtained by the Venn diagram. The heatmap showing the level of common genes was depicted using the R package. According to the receiver operating characteristic (ROC) curves, the area under the curve (AUC) was computed to judge the predictive performance of common genes for RPL patients.

### 2.3 Development and validation of OV prognostic features

The stepwise Akaike information criterion (stepAIC) method from the MASS package V26 was adopted to refine the prognostic genes and build a prognostic model. Besides, the risk score for each patient was calculated according to the normalized levels of the candidate genes (Expi) and their corresponding regression coefficients (Coei) as follows:


$$\text{Risk score}=\sum_{i=1}^{N}\left(Expi\times Coei\right)$$

OV patients were assigned into HR and LR groups based on the median cutoff value. Then, the prognostic value was evaluated through Kaplan–Meier and ROC curve analyses with the ‘survminer’, ‘survival’, and ‘survivalROC’ R packages. Afterward, the prognostic independence of risk score and other clinical indexes in OV patients were estimated via univariate and multivariate Cox analyses.

### 2.4 Prognostic features of the tumor microenvironment

To estimate the TME composition, the enrichment of tumor-infiltrating immune cells was assessed by the R script ssGSEA (single-sample gene set enrichment analysis). Immune checkpoint inhibitors are antitumor immunotherapies that are increasingly used in clinical practice. The immune checkpoint-related gene expression matrix was extracted for differentially expressed gene (DEG) analysis. The tumor immune dysfunction and exclusion (TIDE) score was assessed using the online server (http://tide.dfci.harvard.edu) to determine the effectiveness of immunotherapies in different risk groups.

### 2.5 Stemness signature analysis

First, 26 stemness genes were recruited from StemChecker (http://stemchecker.sysbiolab.eu/), based on the most comprehensive and updated published stemness signatures defined by RNAi screens, gene expression profiles, transcription factor (TF) target gene sets, reports, and computational summary. Then, the stemness enrichment scores of these 26 genes were quantitatively analyzed using ssGSEA via GSVA R package and DEG analysis in both groups.

### 2.6 Functional enrichment analysis

DEGs between the HR and LR groups were first clarified by| logFC | > 0.5 and P < 0.05 for GO analysis using the ‘clusterProfiler’ R package. GSEA of the KEGG pathway between the two groups was implemented using the “clusterProfiler” R package, with |NES| > 1, NOM p-value < 0.05, and q-value < 0.25 as the threshold for enriched items and pathways.

### 2.7 Statistical analysis

R package 4.3.1, 64-bit6 was applied for all analyses. Prognosis and OS were compared using the Kaplan-Meier method and the log-rank test. Kaplan-Meier is a single-factor survival analysis. It is used to study the effect of one factor on survival time and is widely used in the medical field.The continuous variables between two groups were compared using the nonparametric Wilcoxon rank sum test, while comparisons among multiple groups were analyzed using the Kruskal-Wallis test. The Kruskal-Wallis test is a non-parametric test based on ranks, which does not require the original distribution of the sample. Its purpose is to test whether the median of each group is the same.Clinical features with prognostic values were identified through Univariate and multivariate Cox (R package “survival”) analyses.

## 3 Results

### 3.1 Identification of common genes associated with RPL and OV

**Figure 1:**
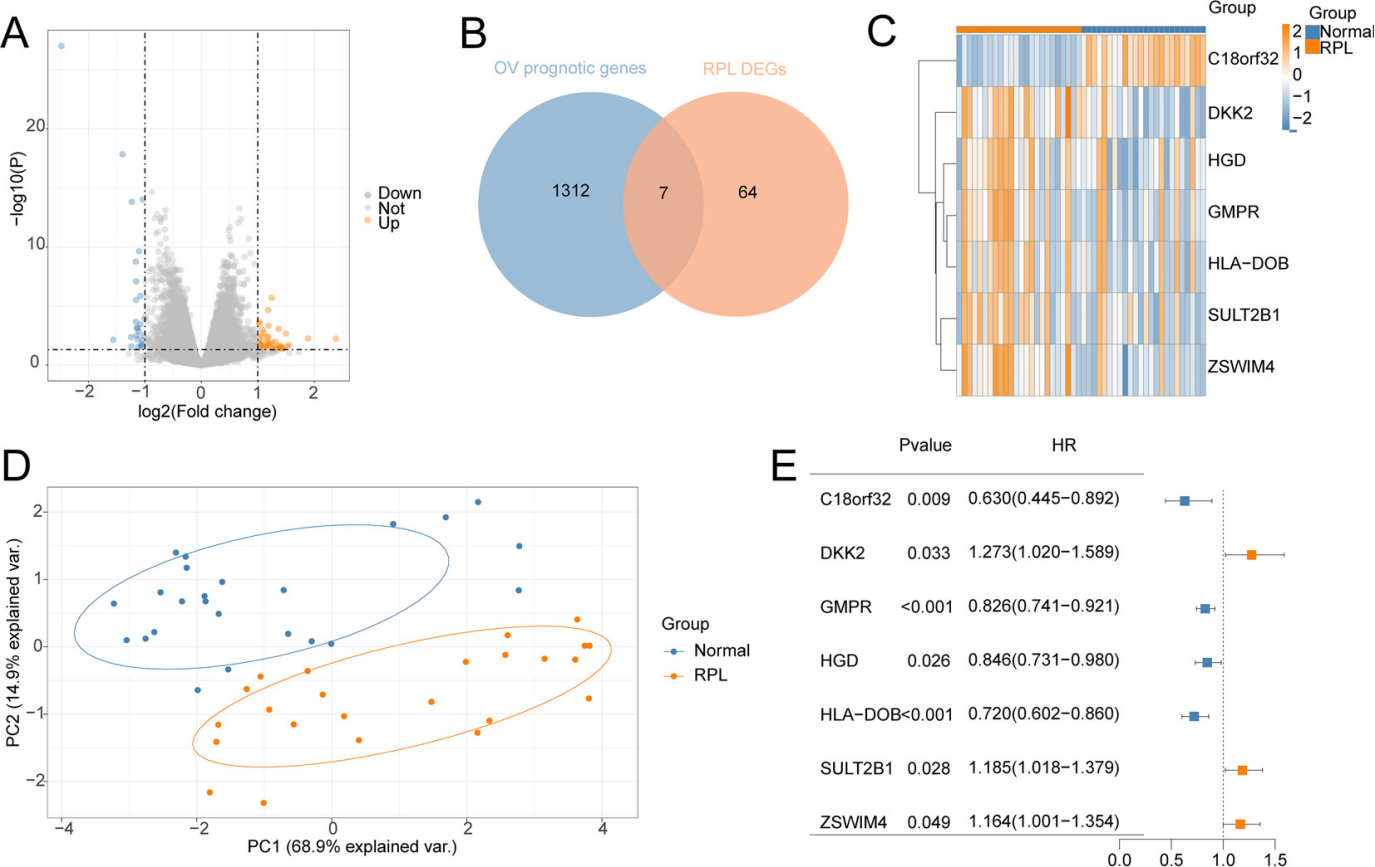
Identification of the common genes between RPL and OV. **(A)** Volcano plot of DEGs in GSE165004 cohort; **(B)** The intersection of OV survival-related genes and RPL-related DEGs; **(C)** Heat map of levels of common genes in RPL samples; **(D)** Principal component analysis of RPL; **(E)** Forest plot of common genes of univariate Cox analysis in OV.

**Figure 2:**
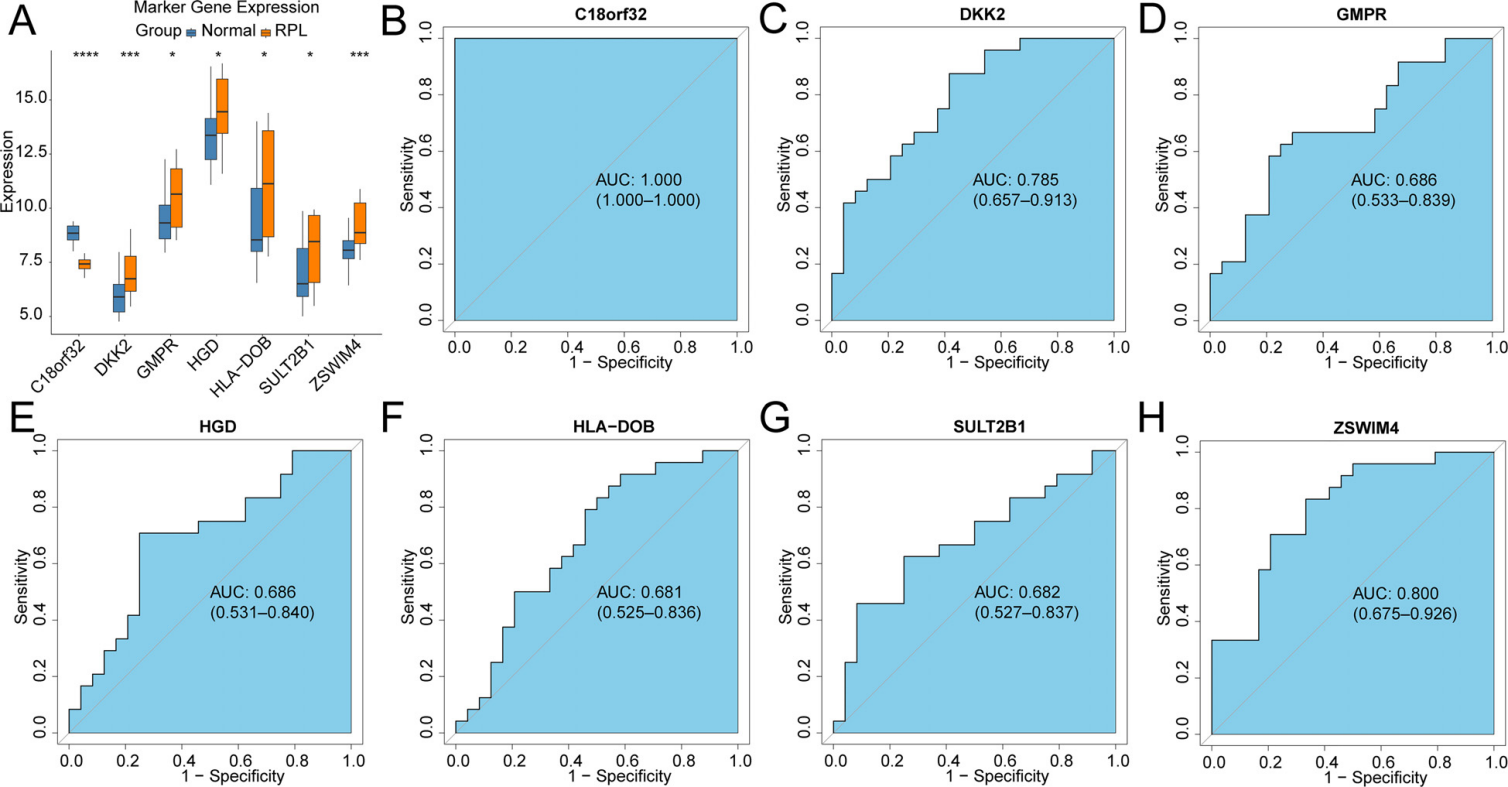
Validation of the diagnostic efficacy based on common genes in the GSE165004 cohort **(A)** Boxplots illustrating the expression differences in common genes between RPL and normal samples. *p < 0.05, ***p < 0.001, ns, no significance; ROC curves of the diagnostic performance of signature genes: **(B)**C18orf32, **(C)** DKK2, **(D)** GMPR, **(E)** HGD, **(F)** HLA-DOB, **(G)** SULT2B1, **(H)** ZSWIM4.

**Figure 3:**
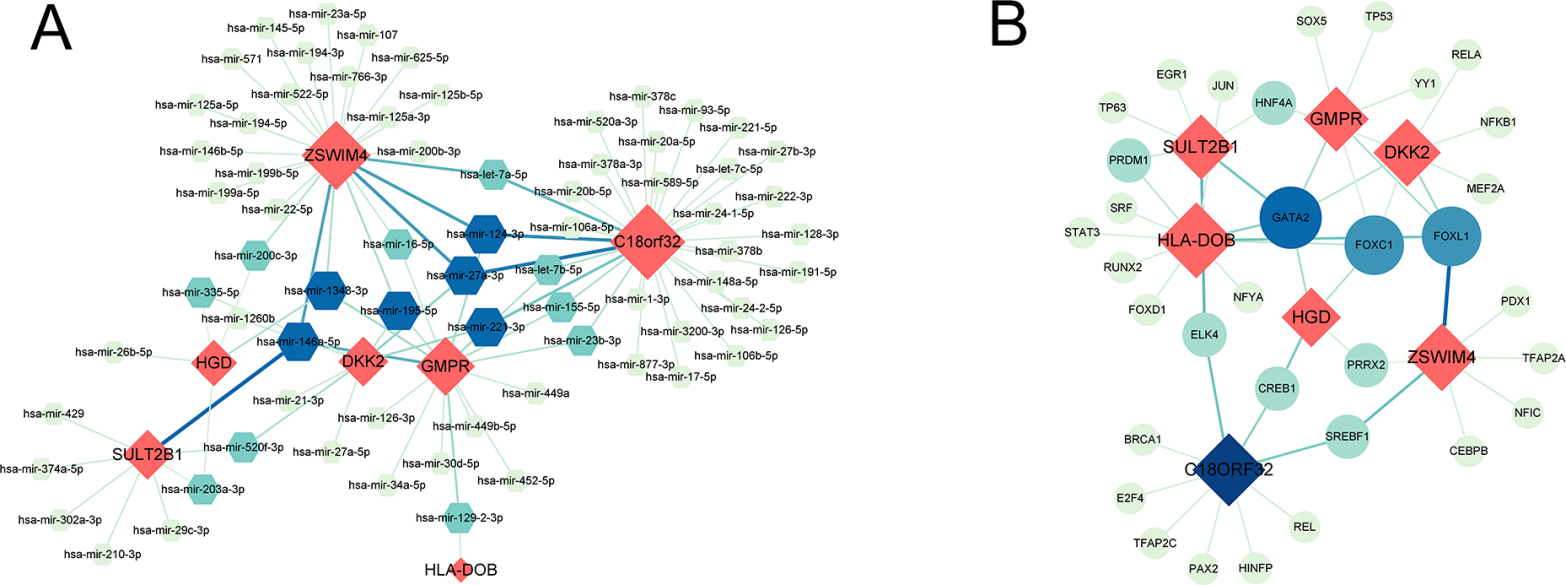
**(A)** Interaction plots of common genes-miRNA. **(B)** Interaction plots of common genes-TF. Red represents common genes.

71 DEGs between RPL and normal tissues of the GSE165004 cohort were identified (**Figure 1A**), while 1427 survival-related genes were identified from univariate Cox analysis of the TCGA-OV cohort (**Supplementary Table S1**). Then the common genes that overlapped from the OV survival-related genes and RPL-related DEGs were determined, and 7 overlapped genes were found, indicating that they were associated with RPL and OV (**Figure 1B**). The expression and Cox regression results of each of the 7 common genes in the GSE165004 and TCGA-OV cohorts are shown in **Figures 1C-E**. In the GSE165004 cohort, C18orf32 level was lower, while DKK2, GMPR, HGD, HLA−DOB, SULT2B1, and ZSWIM4 levels were higher in RPL tissues than in normal controls (**Figure 2A**). Then the diagnostic performance in GSE165004 was estimated. The AUC value was 1.000 for C18orf32 (**Figure 2B**), 0.785 for DKK2 (**Figure 2C**), 0.686 for GMPR (**Figure 2D**), 0.686 for HGD (**Figure 2E**), 0.681 for HLA−DOB (**Figure 2F**), 0.682 for SULT2B1 (**Figure 2G**), and 0.800 for ZSWIM4 (**Figure 2H**). Common genes–miRNA and Common genes–TF regulatory network is also generated using NetworkAnalyst 3.0. The Common genes–miRNA regulatory network comprised 79 nodes and 94 edges (**Figure 3A**), while the Common genes–TF regulatory network comprised 40 nodes and 49 edges (**Figure 3B**) (**Supplementary Table S2**).

### 3.2 Construction and validation of OV prognostic model

**Figure 4:**
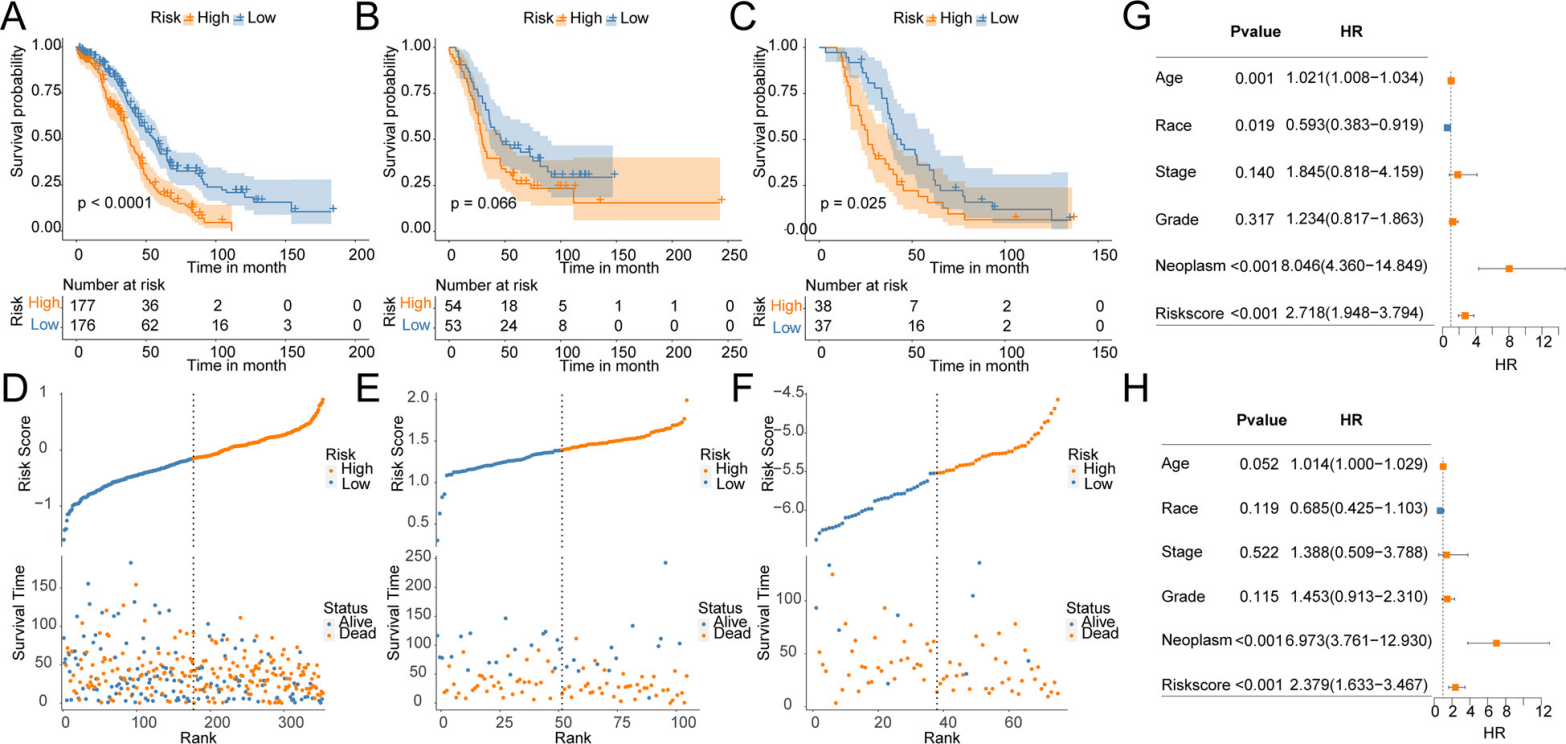
Construction and validation of OV prognostic model. Survival curves of the risk stratification performance of TCGA and GEO cohorts: **(A)** TCGA, **(B)** GSE26193, **(C)** GSE63885. Risk plots of OS: **(D)** TCGA, **(E)** GSE26193, **(F)** GSE63885. Univariate **(G)** and multivariate **(H)** Cox analyses for the prognostic signature and clinical features in the TCGA cohort.

To construct a prognostic model for OV patients, we performed stepAIC Cox analysis from 7 common genes to simplify the model and decrease the number of genes, which yielded a final set of 5 prognostic genes. The 5-gene prognostic model was defined as follows: Risk score = (-0.1510) * GMPR + (-0.2183) * HGD + (-0.2604) * HLA-DOB+ (0.1824) * SULT2B1 + (0.2045) * ZSWIM4. According to the median value, OV patients were stratified into HR (n = 177) and LR (n = 176) groups. Notably, the LR group in the TCGA cohort had higher OS(56.3 months) than the HR group (37.4 months) (P < 0.0001, **Figure 4A**). Additionally, the distribution of risk scores and OS showed that the higher the risk of the patients, the worse the OS rate (**Figure 4D**). To verify the robustness of the model, we used an independent validation group, the TCGA-OV cohort. In GSE26193 and GSE63885 cohorts, patients with LR scores showed better OS than those in the HR group (GSE26193: median time = 46.5 months vs. 29.5 months, P = 0.066, **Figure 4B**; GSE63885: median time = 43.9 months vs. 26.2 months, P = 0.025, **Figure 4C**). The distribution of risk scores and OS in the GSE26193 and GSE63885 cohorts is shown in **Figures 4E, F**. These results validate the robust performance of the model in predicting the prognosis of OV patients in multiple datasets. Univariate and multivariate Cox analyses unveiled that compared with Age, Race, Stage, Grade, and Neoplasm, the risk score was an independent prognostic index for OV patients (**Figures 4G, H**). Collectively, in the TCGA-OV cohort, univariate and multivariate Cox analyses identify risk score as a prognostic index independent of other clinical characteristics.

### 3.3 Association between cancer hallmarks and risk groups

**Figure 5:**
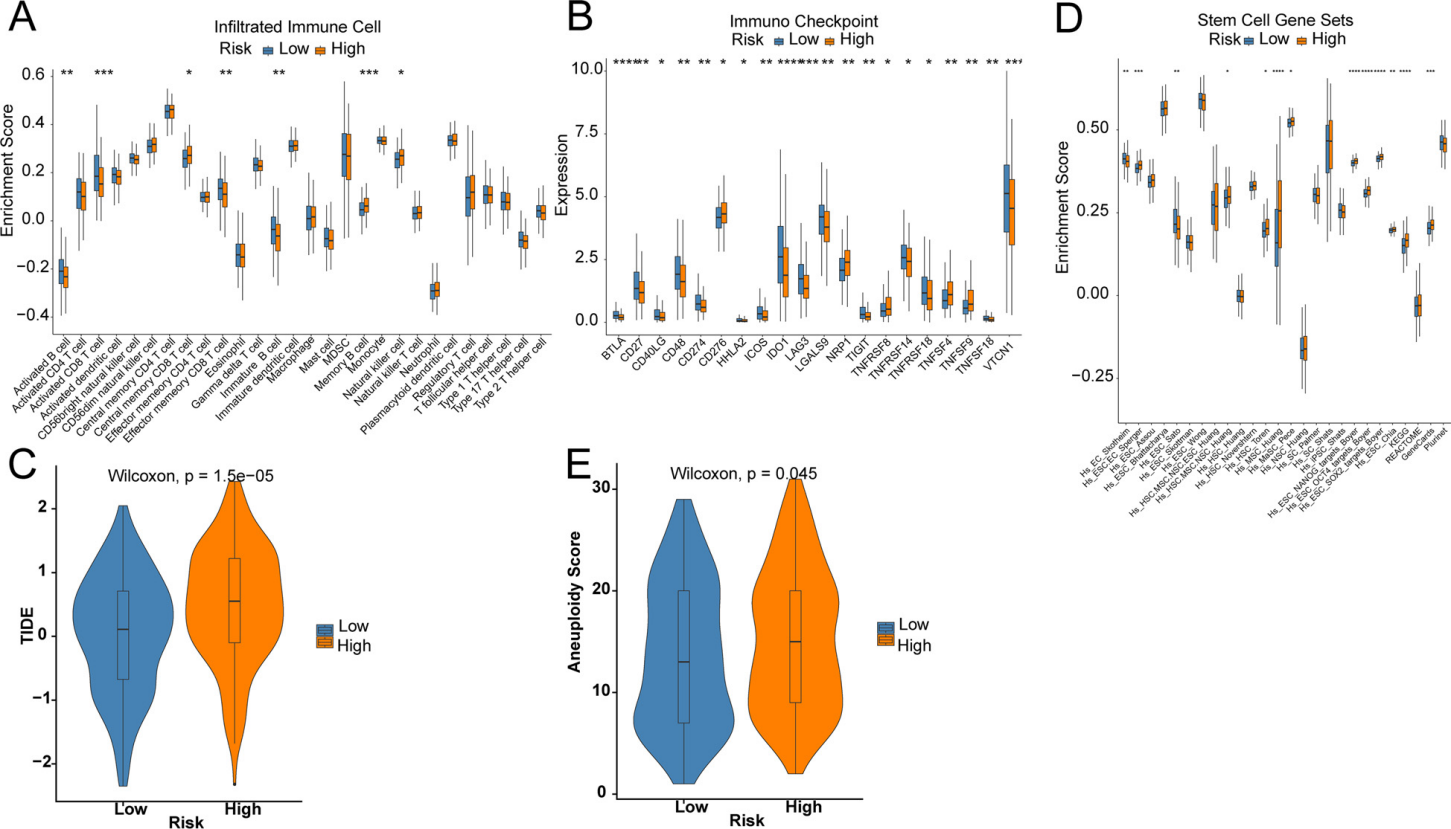
Prediction of the TME and immune cell infiltration. **(A)** Differences in immune cell infiltration levels; **(B)** Differences in immune checkpoint; **(C)** TIDE score; **(D)** Differences in 26 ssGSEA stemness scores; **(E)** Tumor aneuploidy score. Star means significant of difference. The more stars there are, the more significant the difference.

To unveil the association between the risk score and immune cells, and their functions, “ssGSEA” was utilized to test the enrichment scores of immune cell subgroups, related activities, or pathways. Activated CD8 T cells, Activated B cells, immature B cells, and Effector memory CD8 T cells were abundantly infiltrated in the LR group (**Figure 5A**). In addition, the HR group showed higher levels of Central memory CD4 T cells, Memory B cells, and Natural killer cells (**Figure 5A**). Immune checkpoint inhibitors are antitumor immunotherapies that are increasingly used in clinical practice. CD27, CD274, and IDO1 levels were markedly higher in LR patients, while CD276, NRP1, TNFRSF8, and TNFSF4 were greatly higher in HR patients (**Figure 5B**). Furthermore, the TIDE score in LR patients was markedly lower than that in HR patients, suggesting better efficacy of immunotherapy (**Figure 5C**). Even though most OV patients initially respond very well to platinum therapy, tumors eventually show increasing resistance to the treatment. Tumor stem cells are a subgroup of tumor cells, similar to normal stem cells in unlimited proliferation, self-renewal, and multi-directional differentiation. Compared to other tumor cells, cancer stem cells exhibit stronger drug resistance and viability and can evade control and elimination of non-targeted treatment methods, thereby leading to tumor recurrence and metastasis. There was a significantly higher enrichment score of stemness in the HR group by 26 stemness gene sets (**Figure 5D**). The tumor aneuploidy score was notably higher in the HR group (**Figure 5E**).

### 3.4 Identification of prognostic signature-related biological functions

**Figure 6:**
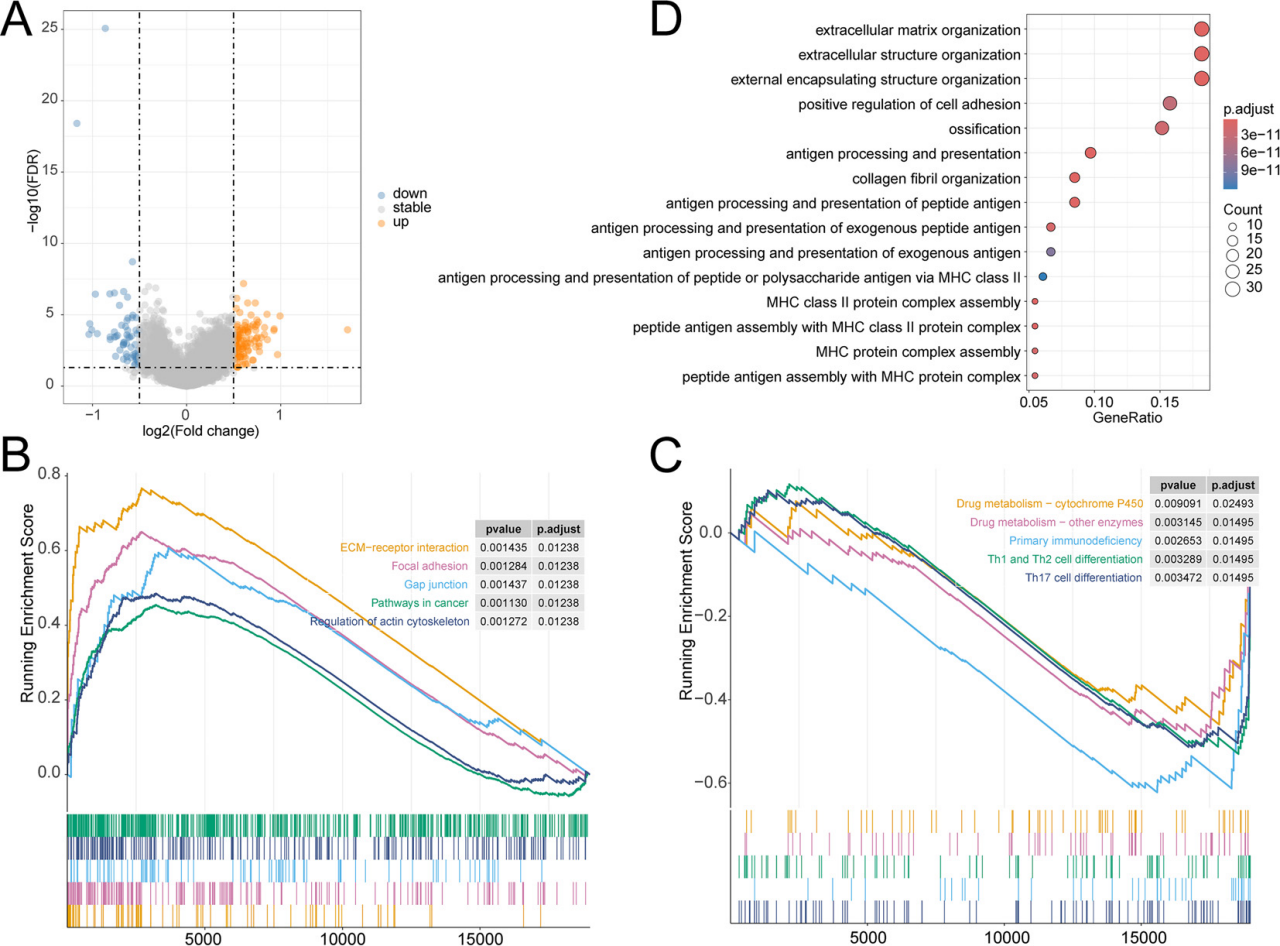
**(A)** Volcano plot of DEGs in TCGA-OV cohort; GSEA analysis of the differential enrichment of KEGG pathways in **(B)** HR group, **(C)** LR group; **(D)** GO analysis of DEGs.

The prognosis of OV has always been a focus of our attention. We have explored the prognostic characteristics of OV and its biological features. The volcano plot visually represented 172 DEGs (**Figure 6A**). The GSEA analysis demonstrated that pathways related to Drug metabolism − cytochrome P450, Drug metabolism − other enzymes, Primary immunodeficiency, Th1 and Th2 cell differentiation, and Th17 cell differentiation were predominantly enriched in the LR group, while ECM−receptor interaction, Focal adhesion, Gap junction, Pathways in cancer, and Regulation of actin cytoskeleton were substantially enriched in the HR group (**Figures 6B, C**). GO analysis noticed that these genes were closely involved in the biological process of extracellular structure organization, positive regulation of cell adhesion, extracellular matrix organization, external encapsulating structure organization, antigen processing and presentation, antigen processing and presentation of peptide antigen, and peptide antigen assembly with MHC class II protein complex (**Figure 6D**).

## 4 Discussion

OV is the second most prevailing gynecological malignancy, with an unclear etiology that may be related to genetic factors, continuous ovulation, and endometriosis. Due to its high recurrence and metastasis rates, the clinical prognosis is extremely poor, troubling both doctors and patients. There are many types of OV, and identifying new predictive biomarkers for OV is of great importance for improving the prognosis of patients. OV is closely related to women’s reproductive conditions. Multiple childbirths are associated with a reduced incidence of OV. Braem et al. discovered the association between RPL and epithelial OV in a prospective study (11). Given the correlation between women’s reproductive conditions and the occurrence of OV, hormonal changes during the reproductive process have attracted increasing attention. The hormonal changes in women’s bodies are related to the onset and progression of OV (10, 12). Here, we developed a new prognostic model for OV based on TCGA-OV and validated its robustness through internal validation cohort within TCGA and external GEO cohorts (GSE26193 and GSE63885). ROC analysis noted the predictive ability of the 7 genes for RPL patients. Then stepAIC analysis was performed to simplify the model and decrease the number of genes, which yielded a final set of 5 prognostic genes with coefficients to construct a prognostic risk scoring system. The risk score calculated using the LASSO algorithm effectively predicted the prognosis of OV patients. Both univariate and multivariate Cox analyses identified risk score as an important prognostic index independent of age, ethnicity, stage, grade, and tumor presence. In both the training and validation cohorts, OV patients in the LR group exhibited longer OS and better prognosis. The above results suggest that our OV risk scoring system has certain clinical values, providing guidance for doctors and patients.

In this work, five common genes, GMPR, HGD, HLA-DOB, SULT2B1, and ZSWIM4, together constituted a stable prognostic risk scoring system for OV. Previous studies have identified GMPR as a potential drug target (13). Zhang et al. constructed a prognostic model of 10 gene components, including GMPR, to discuss the implications for OV immunity and therapy (14). GMPR is gradually increased in Alzheimer’s disease and has the potential as a therapeutic target (15). Heterozygous mutation in GMPR is a pivotal but rare cause of progressive external ophthalmoplegia (PEO) and GMPR is the 19th locus for PEO (16). As a biomarker (KIRC), HGD links to the outcomes of renal clear cell carcinoma and provides theoretical support for the diagnosis and treatment (17). HLA-DOB is closely related to the prognosis of OV, serving as a potential prognostic biomarker for OV (18). HLA-DOB also shows the most prominent differences in gene expression between multiple sclerosis patients and healthy controls (19). SULT2B1 is a novel marker of metastatic colon cancer and is associated with poor prognosis (20). SULT2B1 can indicate inflammatory status, providing insights into potential treatment strategies for atherosclerosis (21). Studies have shown that SULT2B1 silencing inhibits OC progression by targeting ANXA9 (22). ZSWIM4 inhibition enhances the chemotherapy sensitivity of epithelial OV cells by ameliorating intracellular glycine metabolism reprogramming (23).

The predominant type of OV is epithelial OV. The first-line treatment is surgery combined with chemotherapy, and the main chemotherapy regimen is a combination of platinum-based drugs and taxanes. After chemotherapy, targeted administration of olaparib and niraparib can improve the OS as much as possible (4). However, the aggressive and heterogeneous nature of OV contribute to low OS rates and high recurrence rates in many patients. There is a lack of reliable second-line treatment options for frequent relapses (24). Similarly, like OV, RPL remains a challenging condition despite medical advancements. Currently, there are no effective approaches or medications for these conditions. The prognosis of OV is poor, and RPL patients continue to face obstacles in achieving their dreams of motherhood, resulting in potential marital breakdown, career difficulties, depression, and other issues. In recent years, immunotherapy has gained increasing attention from the public, and immunity has emerged as a promising option for many diseases, offering strategies for solving medical challenges and controlling or even curing diseases (25).

Immunomodulatory therapies selectively target immunosuppressive cells in the tumor microenvironment, allowing the activation and proliferation of tumor-specific T cells to identify and eliminate cancer cells (26). Dysregulation of Th cell immunity during pregnancy may lead to recurrent pregnancy loss (RPL) (27).

The TME encompasses surrounding blood vessels, immune cells, the extracellular matrix, fibroblasts, and signaling molecules. The tumor is closely related to and constantly interacts with the surrounding microenvironment. Immune cells within the TME can affect the growth and evolution of cancer cells. Currently, immunoinfiltration therapy, which can generate anti-tumor immunity, is available for the treatment of cancer. Extensive characterization of the TME is critical to identify effective prognostic markers and immunotherapeutic targets for OV. The clinical application of molecularly targeted therapies is evolving rapidly, but it primarily focuses on genomic alterations. The TME is complex and diverse, and understanding the specific immune TME is very important for predicting the effectiveness of immunotherapies. As a key mediator, the TME allows patients with different types of tumors to achieve significant clinical effects after immunotherapy (28). The immune system can influence tumor growth and mutation, generating anti-tumor immunity, while tumor cells can also damage immune cells in various ways. Besides, OV patients have poor prognoses due to increased TET3 expression, low BCL7A expression, increased Thy-1 expression, and hypermethylation of tumor suppressor genes (29–32). MiRNA molecules are single-stranded RNA molecules, and the dysregulation of MiRNA expression is related to pathological processes such as RPL, and more than 60 mirnas are related to the occurrence and development of ovarian cancer (33–35).

In the TCGA-OV cohort, we developed a prognostic scoring system for OV, as well as predictive models for immunotherapy and immune infiltration. With these models, OV patients were classified into HR and LR groups. Ultimately, it was found that the LR patients had significantly better outcomes than the HR individuals in terms of immunotherapy effectiveness and prognosis. The prognosis of OV patients can be precisely predicted through our prognostic scoring system, which can provide personalized treatment and prolong the survival of the patients. Currently, in addition to targeted therapy (PARP inhibitors, anti-angiogenesis inhibitors), immunotherapy is also a novel treatment regimen for OV. However, existing research results indicate that immunotherapy provides minimal benefits for individuals with advanced or recurrent OV. At present, there are no FDA-approved immunotherapy drugs for OV. Although immunotherapy may be the most effective treatment for RPL, its clinical application is hindered due to limited research. Our molecular studies provide references for future research to explore the molecular mechanisms and pathways more closely associated with RPL and OV, and to formulate tailored and accurate treatment strategies, bringing hope to patients with both conditions.

## Data Availability

The original contributions presented in the study are included in the article/**Supplementary Material**. Further inquiries can be directed to the corresponding author.

## References

[1] Bray F Ferlay J Soerjomataram I Siegel RL Torre LA Jemal A. Global cancer statistics 2018: GLOBOCAN estimates of incidence and mortality worldwide for 36 cancers in 185 countries. CA Cancer J Clin. (2018) 68:394–424. doi: 10.3322/caac.21492 30207593

[2] Torre LA Trabert B DeSantis CE Miller KD Samimi G Runowicz CD. Ovarian cancer statistics, 2018. CA Cancer J Clin. (2018) 68:284–96. doi: 10.3322/caac.21456 PMC6621554 29809280

[3] Kindler O Pulkkinen O Cherstvy AG Metzler R. Burst statistics in an early biofilm quorum sensing model: the role of spatial colony-growth heterogeneity. Sci Rep. (2019) 9:12077. doi: 10.1038/s41598-019-48525-2 31427659 PMC6700081

[4] Moufarrij S Dandapani M Arthofer E Gomez S Srivastava A Lopez-Acevedo M. Epigenetic therapy for ovarian cancer: promise and progress. Clin Epigenetics. (2019) 11:7. doi: 10.1186/s13148-018-0602-0 30646939 PMC6334391

[5] Practice Committee of the American Society for Reproductive Medicine. Evaluation and treatment of recurrent pregnancy loss: a committee opinion. Fertil Steril. (2012) 98:1103–11. doi: 10.1016/j.fertnstert.2012.06.048 22835448

[6] Bender Atik R Christiansen OB Elson J Kolte AM Lewis S Middeldorp S. ESHRE guideline: recurrent pregnancy loss. Hum Reprod Open. (2018) 2018:hoy004. doi: 10.1093/hropen/hoy004 31486805 PMC6276652

[7] Alijotas-Reig J Garrido-Gimenez C. Current concepts and new trends in the diagnosis and management of recurrent miscarriage. Obstet Gynecol Surv. (2013) 68:445–66. doi: 10.1097/OGX.0b013e31828aca19 23942472

[8] van Dijk MM Kolte AM Limpens J Kirk E Quenby S van Wely M. Recurrent pregnancy loss: diagnostic workup after two or three pregnancy losses? A systematic review of the literature and meta-analysis. Hum Reprod Update. (2020) 26:356–67. doi: 10.1093/humupd/dmz048 PMC7161667 32103270

[9] Wu AH Pearce CL Lee AW Tseng C Jotwani A Patel P. Timing of births and oral contraceptive use influences ovarian cancer risk. Int J Cancer. (2017) 141:1–21. doi: 10.1002/ijc.30910 PMC7560976 28748634

[10] Lukanova A Kaaks R. Endogenous hormones and ovarian cancer: epidemiology and current hypotheses. Cancer Epidemiol Biomarkers Prev. (2005) 14:98–107. doi: 10.1158/1055-9965.98.14.1 15668482

[11] Braem MG Onland-Moret NC Schouten LJ Kruitwagen RF Lukanova A Allen NE. Multiple miscarriages are associated with the risk of ovarian cancer: results from the European Prospective Investigation into Cancer and Nutrition. PloS One. (2012) 7:e37141. doi: 10.1371/journal.pone.0037141 22623987 PMC3356371

[12] Huang Z Beeghly-Fadiel A Gao YT Zheng Y Dai Q Lu W. Associations of reproductive time events and intervals with breast cancer risk: a report from the Shanghai Breast Cancer Study. Int J Cancer. (2014) 135:186–95. doi: 10.1002/ijc.v135.1 PMC4591050 24323821

[13] Gaiya DD Muhammad A Aimola IA Udu SK Balarabe SA Auta R. Potential of Onchocerca ochengi inosine-5'-monophosphate dehydrogenase (IMPDH) and guanosine-5'-monophosphate oxidoreductase (GMPR) as druggable and vaccine candidates: immunoinformatics screening. J Biomol Struct Dyn. (2023) 41:14832–48. doi: 10.1080/07391102.2023.2184171 36866624

[14] Zhang X Han L Zhang H Niu Y Liang R. Identification of potential key genes of TGF-beta signaling associated with the immune response and prognosis of ovarian cancer based on bioinformatics analysis. Heliyon. (2023) 9:e19208. doi: 10.1016/j.heliyon.2023.e19208 37664697 PMC10469581

[15] Liu H Luo K Luo D. Guanosine monophosphate reductase 1 is a potential therapeutic target for Alzheimer's disease. Sci Rep. (2018) 8:2759. doi: 10.1038/s41598-018-21256-6 29426890 PMC5807363

[16] Sommerville EW Dalla Rosa I Rosenberg MM Bruni F Thompson K Rocha M. Identification of a novel heterozygous guanosine monophosphate reductase (GMPR) variant in a patient with a late-onset disorder of mitochondrial DNA maintenance. Clin Genet. (2020) 97:276–86. doi: 10.1111/cge.13652 PMC7004030 31600844

[17] Wang J Chang H Su M Qiao Y Sun H Zhao Y. Identification of HGD and GSTZ1 as biomarkers involved metabolic reprogramming in kidney renal clear cell carcinoma. Int J Mol Sci. (2022) 23:4583. doi: 10.3390/ijms23094583 35562974 PMC9102202

[18] Li Y Li H Yang B Wei J Zhen C Feng L. Clinical significance of PI3 and HLA-DOB as potential prognostic predicators for ovarian cancer. Transl Cancer Res. (2020) 9:466–76. doi: 10.21037/tcr.2019.11.30 PMC8798007 35117391

[19] Li H Hou X Liang Y Xu F Zhang X Cui P. Gene-based tests of a genome-wide association study dataset highlight novel multiple sclerosis risk genes. Front Neurosci. (2021) 15:614528. doi: 10.3389/fnins.2021.614528 34045940 PMC8144314

[20] Che G Wang W Wang J He C Yin J Chen Z. Sulfotransferase SULT2B1 facilitates colon cancer metastasis by promoting SCD1-mediated lipid metabolism. Clin Transl Med. (2024) 14:e1587. doi: 10.1002/ctm2.v14.2 38372484 PMC10875708

[21] Pan H Wu T Huang K Guo Z Liang H Lyu P. Reducing SULT2B1 promotes the interaction of LncRNAgga3-204 with SMAD4 to inhibit the macrophage inflammatory response and delay atherosclerosis progression. Transl Res. (2024) 268:13–27. doi: 10.1016/j.trsl.2024.01.004 38286358

[22] Gao H Xia M Ruan H. Knockdown of sulfotransferase 2B1 suppresses cell migration, invasion and promotes apoptosis in ovarian carcinoma cells via targeting annexin A9. J Obstet Gynaecol Res. (2024) 50:1334–44. doi: 10.1111/jog.15969 38777329

[23] Gong K Huang Y Zheng Y Hao W Shi K. ZSWIM4 inhibition improves chemosensitivity in epithelial ovarian cancer cells by suppressing intracellular glycine biosynthesis. J Transl Med. (2024) 22:192. doi: 10.1186/s12967-024-04980-8 38383406 PMC10880229

[24] Wefers C Lambert LJ Torensma R Hato SV. Cellular immunotherapy in ovarian cancer: Targeting the stem of recurrence. Gynecol Oncol. (2015) 137:335–42. doi: 10.1016/j.ygyno.2015.02.019 25727651

[25] Zhang Y Zhang Z. The history and advances in cancer immunotherapy: understanding the characteristics of tumor-infiltrating immune cells and their therapeutic implications. Cell Mol Immunol. (2020) 17:807–21. doi: 10.1038/s41423-020-0488-6 PMC7395159 32612154

[26] Siminiak N Czepczyński R Zaborowski MP Iżycki D. Immunotherapy in ovarian cancer. Arch Immunol Ther Exp (Warsz). (2022) 70:19. doi: 10.1007/s00005-022-00655-8 35941287 PMC9360103

[27] Wang W Sung N Gilman-Sachs A Kwak-Kim J. T helper (Th) cell profiles in pregnancy and recurrent pregnancy losses: th1/th2/th9/th17/th22/tfh cells. Front Immunol. (2020) 11:2025. doi: 10.3389/fimmu.2020.02025 32973809 PMC7461801

[28] Bagaev A Kotlov N Nomie K Svekolkin V Gafurov A Isaeva O. Conserved pan-cancer microenvironment subtypes predict response to immunotherapy. Cancer Cell. (2021) 39:845–65.e7. doi: 10.1016/j.ccell.2021.04.014 34019806

[29] Cao T Pan W Sun X Shen H. Increased expression of TET3 predicts unfavorable prognosis in patients with ovarian cancer-a bioinformatics integrative analysis. J Ovarian Res. (2019) 12:101. doi: 10.1186/s13048-019-0575-4 31656201 PMC6816171

[30] Sun Z Sun L He M Pang Y Yang Z Wang J. Low BCL7A expression predicts poor prognosis in ovarian cancer. J Ovarian Res. (2019) 12:41. doi: 10.1186/s13048-019-0518-0 31077237 PMC6511192

[31] Connor EV Saygin C Braley C Wiechert AC Karunanithi S Crean-Tate K. Thy-1 predicts poor prognosis and is associated with self-renewal in ovarian cancer. J Ovarian Res. (2019) 12:112. doi: 10.1186/s13048-019-0590-5 31735168 PMC6858973

[32] Feng LY Chen CX Li L. Hypermethylation of tumor suppressor genes is a risk factor for poor prognosis in ovarian cancer: A meta-analysis. Med (Baltimore). (2019) 98:e14588. doi: 10.1097/MD.0000000000014588 PMC6408028 30813180

[33] Santamaria X Taylor H. MicroRNA and gynecological reproductive diseases. Fertil Steril. (2014) 101:1545–51. doi: 10.1016/j.fertnstert.2014.04.044 24882618

[34] Wang X Ivan M Hawkins SM. The role of MicroRNA molecules and MicroRNA-regulating machinery in the pathogenesis and progression of epithelial ovarian cancer. Gynecol Oncol. (2017) 147:481–7. doi: 10.1016/j.ygyno.2017.08.027 PMC5741188 28866430

[35] Chong ZX. Roles of miRNAs in regulating ovarian cancer stemness. Biochim Biophys Acta Rev Cancer. (2024) 1879:189191. doi: 10.1016/j.bbcan.2024.189191 39353485

