# Supplementary material for: Potential shared gene signatures and molecular mechanisms between recurrent pregnancy loss and ovarian cancer
Source: Front Oncol. 2024 Nov 14;14:1445502. doi: 10.3389/fonc.2024.1445502 (PMC11602398; doi:10.3389/fonc.2024.1445502)
Supplement: Supplementary file 1 [file Table1.docx]

**Supplementary Table S1** 1427 survival-related genes were identified from univariate Cox regression analysis of the TCGA-OV cohort.

| Table S1 |
| --- |
| ZNF90 |
| CAPN6 |
| SLC7A4 |
| PDZK1 |
| SCGB1D4 |
| DUOXA1 |
| PNMT |
| LRRC26 |
| HLA-DOB |
| KCNG1 |
| CLDN24 |
| C18orf32 |
| TM4SF4 |
| WNK4 |
| ADAMTS8 |
| CTNNA2 |
| KCNK13 |
| CHST4 |
| PLA2G4D |
| TNNC1 |
| ALPL |
| RAMP2 |
| IGLL5 |
| EFEMP1 |
| PGAM4 |
| MAP2K6 |
| SCGB1D2 |
| PPP1R1B |
| CWH43 |
| PAPSS1 |
| FOLR1 |
| FAM107A |
| PDE6A |
| SURF4 |
| GJA1 |
| GNG2 |
| SLC43A3 |
| PLA2G4F |
| HAPLN1 |
| INHBA |
| PPP6R1 |
| TNFAIP6 |
| SST |
| PCDH17 |
| MCOLN2 |
| SUGT1 |
| ENTPD8 |
| PCSK6 |
| IGSF9 |
| OAS1 |
| CFB |
| ZNF750 |
| SIDT1 |
| CNN1 |
| GALNT14 |
| SULT2B1 |
| CLU |
| HGD |
| CPZ |
| SULT1E1 |
| CD2AP |
| AZGP1 |
| PLD5 |
| FMO5 |
| CTSZ |
| DKK2 |
| ZSWIM4 |
| ALDH3B2 |
| LCN12 |
| ASPN |
| GMPR |

**Supplementary Table S2** The Common genes–miRNA regulatory network comprises 79 nodes and 94 edges, while Common genes–TF regulatory network comprises 40 nodes and 49 edges.

| AverageShortestPathLength | BetweennessCentrality | ClosenessCentrality | ClusteringCoefficient | Degree | Eccentricity | IsSingleNode | name | NeighborhoodConnectivity | NumberOfDirectedEdges | NumberOfUndirectedEdges | PartnerOfMultiEdgedNodePairs | Radiality | selected | SelfLoops | shared name | Stress | TopologicalCoefficient | type |
| --- | --- | --- | --- | --- | --- | --- | --- | --- | --- | --- | --- | --- | --- | --- | --- | --- | --- | --- |
| 3.666666667 | 0 | 0.272727273 | 0 | 1 | 6 | FALSE | FOXD1 | 10 | 0 | 1 | 0 | 0.733333333 | FALSE | 0 | FOXD1 | 0 | 0 | gene |
| 3.666666667 | 0 | 0.272727273 | 0 | 1 | 6 | FALSE | JUN | 10 | 0 | 1 | 0 | 0.733333333 | FALSE | 0 | JUN | 0 | 0 | gene |
| 2.692307692 | 0.098610308 | 0.371428571 | 0 | 4 | 4 | FALSE | FOXC1 | 6.75 | 0 | 4 | 0 | 0.830769231 | FALSE | 0 | FOXC1 | 506 | 0.319444444 | gene |
| 2.487179487 | 0.217426579 | 0.402061856 | 0 | 5 | 4 | FALSE | GATA2 | 6.6 | 0 | 5 | 0 | 0.851282051 | FALSE | 0 | GATA2 | 1084 | 0.266666667 | gene |
| 2.487179487 | 0.238075959 | 0.402061856 | 0 | 4 | 4 | FALSE | FOXL1 | 7.5 | 0 | 4 | 0 | 0.851282051 | FALSE | 0 | FOXL1 | 852 | 0.295454545 | gene |
| 3.666666667 | 0 | 0.272727273 | 0 | 1 | 6 | FALSE | NFYA | 10 | 0 | 1 | 0 | 0.733333333 | FALSE | 0 | NFYA | 0 | 0 | gene |
| 3.666666667 | 0 | 0.272727273 | 0 | 1 | 6 | FALSE | SRF | 10 | 0 | 1 | 0 | 0.733333333 | FALSE | 0 | SRF | 0 | 0 | gene |
| 3.666666667 | 0 | 0.272727273 | 0 | 1 | 6 | FALSE | STAT3 | 10 | 0 | 1 | 0 | 0.733333333 | FALSE | 0 | STAT3 | 0 | 0 | gene |
| 3.051282051 | 0.038802134 | 0.327731092 | 0 | 2 | 4 | FALSE | PRDM1 | 8 | 0 | 2 | 0 | 0.794871795 | FALSE | 0 | PRDM1 | 214 | 0.538461538 | gene |
| 3.666666667 | 0 | 0.272727273 | 0 | 1 | 6 | FALSE | RUNX2 | 10 | 0 | 1 | 0 | 0.733333333 | FALSE | 0 | RUNX2 | 0 | 0 | gene |
| 4.128205128 | 0 | 0.242236025 | 0 | 1 | 6 | FALSE | REL | 9 | 0 | 1 | 0 | 0.687179487 | FALSE | 0 | REL | 0 | 0 | gene |
| 4.128205128 | 0 | 0.242236025 | 0 | 1 | 6 | FALSE | BRCA1 | 9 | 0 | 1 | 0 | 0.687179487 | FALSE | 0 | BRCA1 | 0 | 0 | gene |
| 3.153846154 | 0.091285907 | 0.317073171 | 0 | 2 | 4 | FALSE | CREB1 | 6.5 | 0 | 2 | 0 | 0.784615385 | FALSE | 0 | CREB1 | 488 | 0.5 | gene |
| 4.128205128 | 0 | 0.242236025 | 0 | 1 | 6 | FALSE | HINFP | 9 | 0 | 1 | 0 | 0.687179487 | FALSE | 0 | HINFP | 0 | 0 | gene |
| 3 | 0.105489686 | 0.333333333 | 0 | 2 | 4 | FALSE | SREBF1 | 8 | 0 | 2 | 0 | 0.8 | FALSE | 0 | SREBF1 | 364 | 0.5 | gene |
| 4.128205128 | 0 | 0.242236025 | 0 | 1 | 6 | FALSE | TFAP2C | 9 | 0 | 1 | 0 | 0.687179487 | FALSE | 0 | TFAP2C | 0 | 0 | gene |
| 3.051282051 | 0.114182893 | 0.327731092 | 0 | 2 | 4 | FALSE | ELK4 | 7.5 | 0 | 2 | 0 | 0.794871795 | FALSE | 0 | ELK4 | 500 | 0.5 | gene |
| 4.128205128 | 0 | 0.242236025 | 0 | 1 | 6 | FALSE | PAX2 | 9 | 0 | 1 | 0 | 0.687179487 | FALSE | 0 | PAX2 | 0 | 0 | gene |
| 4.128205128 | 0 | 0.242236025 | 0 | 1 | 6 | FALSE | E2F4 | 9 | 0 | 1 | 0 | 0.687179487 | FALSE | 0 | E2F4 | 0 | 0 | gene |
| 3.615384615 | 0 | 0.276595745 | 0 | 1 | 6 | FALSE | PDX1 | 7 | 0 | 1 | 0 | 0.738461538 | FALSE | 0 | PDX1 | 0 | 0 | gene |
| 3.256410256 | 0.017918193 | 0.307086614 | 0 | 2 | 4 | FALSE | PRRX2 | 5.5 | 0 | 2 | 0 | 0.774358974 | FALSE | 0 | PRRX2 | 80 | 0.5 | gene |
| 3.615384615 | 0 | 0.276595745 | 0 | 1 | 6 | FALSE | CEBPB | 7 | 0 | 1 | 0 | 0.738461538 | FALSE | 0 | CEBPB | 0 | 0 | gene |
| 3.615384615 | 0 | 0.276595745 | 0 | 1 | 6 | FALSE | NFIC | 7 | 0 | 1 | 0 | 0.738461538 | FALSE | 0 | NFIC | 0 | 0 | gene |
| 3.615384615 | 0 | 0.276595745 | 0 | 1 | 6 | FALSE | TFAP2A | 7 | 0 | 1 | 0 | 0.738461538 | FALSE | 0 | TFAP2A | 0 | 0 | gene |
| 3.871794872 | 0 | 0.258278146 | 0 | 1 | 6 | FALSE | YY1 | 6 | 0 | 1 | 0 | 0.712820513 | FALSE | 0 | YY1 | 0 | 0 | gene |
| 3.871794872 | 0 | 0.258278146 | 0 | 1 | 6 | FALSE | TP53 | 6 | 0 | 1 | 0 | 0.712820513 | FALSE | 0 | TP53 | 0 | 0 | gene |
| 3.871794872 | 0 | 0.258278146 | 0 | 1 | 6 | FALSE | SOX5 | 6 | 0 | 1 | 0 | 0.712820513 | FALSE | 0 | SOX5 | 0 | 0 | gene |
| 3.820512821 | 0 | 0.261744966 | 0 | 1 | 6 | FALSE | MEF2A | 7 | 0 | 1 | 0 | 0.717948718 | FALSE | 0 | MEF2A | 0 | 0 | gene |
| 3.205128205 | 0.024227235 | 0.312 | 0 | 2 | 4 | FALSE | HNF4A | 6.5 | 0 | 2 | 0 | 0.779487179 | FALSE | 0 | HNF4A | 142 | 0.55 | gene |
| 3.820512821 | 0 | 0.261744966 | 0 | 1 | 6 | FALSE | NFKB1 | 7 | 0 | 1 | 0 | 0.717948718 | FALSE | 0 | NFKB1 | 0 | 0 | gene |
| 3.820512821 | 0 | 0.261744966 | 0 | 1 | 6 | FALSE | RELA | 7 | 0 | 1 | 0 | 0.717948718 | FALSE | 0 | RELA | 0 | 0 | gene |
| 3.769230769 | 0 | 0.265306122 | 0 | 1 | 6 | FALSE | TP63 | 6 | 0 | 1 | 0 | 0.723076923 | FALSE | 0 | TP63 | 0 | 0 | gene |
| 3.769230769 | 0 | 0.265306122 | 0 | 1 | 6 | FALSE | EGR1 | 6 | 0 | 1 | 0 | 0.723076923 | FALSE | 0 | EGR1 | 0 | 0 | gene |
| 2.692307692 | 0.323536405 | 0.371428571 | 0 | 10 | 5 | FALSE | HLA-DOB | 2.1 | 0 | 10 | 0 | 0.830769231 | FALSE | 0 | HLA-DOB | 1308 | 0.22 | core |
| 3.153846154 | 0.316163807 | 0.317073171 | 0 | 9 | 5 | FALSE | C18ORF32 | 1.333333333 | 0 | 9 | 0 | 0.784615385 | FALSE | 0 | C18ORF32 | 1218 | 0.111111111 | core |
| 2.641025641 | 0.278352934 | 0.378640777 | 0 | 7 | 5 | FALSE | ZSWIM4 | 1.714285714 | 0 | 7 | 0 | 0.835897436 | FALSE | 0 | ZSWIM4 | 832 | 0.142857143 | core |
| 2.641025641 | 0.144066898 | 0.378640777 | 0 | 4 | 3 | FALSE | HGD | 3.25 | 0 | 4 | 0 | 0.835897436 | FALSE | 0 | HGD | 726 | 0.375 | core |
| 2.897435897 | 0.160420281 | 0.345132743 | 0 | 6 | 5 | FALSE | GMPR | 2.666666667 | 0 | 6 | 0 | 0.81025641 | FALSE | 0 | GMPR | 624 | 0.333333333 | core |
| 2.846153846 | 0.188313733 | 0.351351351 | 0 | 7 | 5 | FALSE | DKK2 | 2.571428571 | 0 | 7 | 0 | 0.815384615 | FALSE | 0 | DKK2 | 822 | 0.314285714 | core |
| 2.794871795 | 0.219375361 | 0.357798165 | 0 | 6 | 5 | FALSE | SULT2B1 | 2.166666667 | 0 | 6 | 0 | 0.820512821 | FALSE | 0 | SULT2B1 | 932 | 0.233333333 | core |
